# Reinforcement Learning-Based Complete Area Coverage Path Planning for a Modified hTrihex Robot

**DOI:** 10.3390/s21041067

**Published:** 2021-02-04

**Authors:** Koppaka Ganesh Sai Apuroop, Anh Vu Le, Mohan Rajesh Elara, Bing J. Sheu

**Affiliations:** 1 ROAR Lab, Engineering Product Development, Singapore University of Technology and Design, Singapore 487372, Singapore; ganesh_koppaka@mymail.sutd.edu.sg (K.G.S.A.); rajeshelara@sutd.edu.sg (M.R.E.); 2 Optoelectronics Research Group, Faculty of Electrical and Electronics Engineering, Ton Duc Thang University, Ho Chi Minh City 700000, Vietnam; 3 Electronics Engineering and Information Management, Chang Gung University, Taoyuan City 330, Taiwan; bsheu@mail.cgu.edu.tw

**Keywords:** reconfigurable robot, tiling robots, reinforcement learning, complete coverage planing, energy path planning

## Abstract

One of the essential attributes of a cleaning robot is to achieve complete area coverage. Current commercial indoor cleaning robots have fixed morphology and are restricted to clean only specific areas in a house. The results of maximum area coverage are sub-optimal in this case. Tiling robots are innovative solutions for such a coverage problem. These new kinds of robots can be deployed in the cases of cleaning, painting, maintenance, and inspection, which require complete area coverage. Tiling robots’ objective is to cover the entire area by reconfiguring to different shapes as per the area requirements. In this context, it is vital to have a framework that enables the robot to maximize the area coverage while minimizing energy consumption. That means it is necessary for the robot to cover the maximum area with the least number of shape reconfigurations possible. The current paper proposes a complete area coverage planning module for the modified hTrihex, a honeycomb-shaped tiling robot, based on the deep reinforcement learning technique. This framework simultaneously generates the tiling shapes and the trajectory with minimum overall cost. In this regard, a convolutional neural network (CNN) with long short term memory (LSTM) layer was trained using the actor-critic experience replay (ACER) reinforcement learning algorithm. The simulation results obtained from the current implementation were compared against the results that were generated through traditional tiling theory models that included zigzag, spiral, and greedy search schemes. The model presented in the current paper was also compared against other methods where this problem was considered as a traveling salesman problem (TSP) solved through genetic algorithm (GA) and ant colony optimization (ACO) approaches. Our proposed scheme generates a path with a minimized cost at a lesser time.

## 1. Introduction

Cleaning has always been an important activity to humans. Cleaning requires maximum area coverage. Recently, based on the principles of autonomous area coverage, many robots have been deployed in essential life aspects such as underwater operations [1], de-mining [2,3], agriculture [4,5], painting [6], rescue operations [7], tiling robotics [8,9,10], ship hull cleaning [11,12], benchmarking for inspection [13], pavement sweeping [14], and so on. Currently, cleaning robots for domestic and industrial markets are in heavy demand. Over time, the tools required to clean have also evolved. However, most of the cleaning tasks remain manual and laborious. Nowadays, many people either delegate their cleaning activities to others or clean their premises on their own. Hence, there are vast opportunities for automating cleaning tasks, especially in home settings. Indeed, commercial autonomous cleaning devices for houses have seen a rise over the past few years. Even though they are commercially successful, their fixed morphology hinders them from reaching their maximum cleaning capacity. The commonly available commercial cleaning robots are round in shape, and this fixed shape cannot allow them to reach tight spaces under an indoor setting. This impacts the area coverage which is a crucial factor for cleaning robots. Thus, there is a need for robots that can change shape to access tight spaces based on the layout, and that is where reconfigurable tiling robots come into the picture. Reconfigurable tiling robotics is an interesting research topic wherein the robots reconfigure to different shapes to implement applications such as floor-cleaning [15,16], among others. The main objective of the reconfigurable tiling robots is to maximize the area coverage. The ability to change their morphology helps them to achieve this objective. There are several reconfigurable tiling robots developed by researchers from the ROAR laboratory to perform cleaning and other tasks [17,18,19]. The robots mentioned here can morph into different shapes. The robot from [17] can morph into seven shapes, and the robots from [18,19] can morph into three shapes each. In the current study, the modified hTrihex, a honeycomb-shaped tiling robot, was utilized to test our proposed scheme. The ability to reconfigure gives the robot the advantage of maneuvering around the obstacles and also reaching hard-to-clean spaces.

The challenging problem involved with a reconfigurable tiling robot is to create an optimal tiling set and a trajectory that covers the entire area. As we know, the traditional path planning algorithms generate the most efficient paths between the start and the goal locations. In our context, we require a trajectory that covers the entire area. This brought us to implementing the complete area coverage path planning (CACPP) approaches for the current robot. There are two main objectives for the CACPP module: it should generate an energy-efficient path that can help the robot visit every point in the given area while avoiding the obstacles.

CACPP approaches have been implemented on various fixed morphology robots, and one of them was mentioned in [20]. The authors described a complete coverage path planning and guidance methodology for mobile robots to clean large industrial areas. The authors in [21] presented a method for complete area coverage for mobile robots whose applications include de-mining, cleaning, and painting, among others. They implemented a novel cell decomposition algorithm that can divide a given area into several cells covered by the robot’s motion. In paper [22], the authors presented a modified version of the A* algorithm; the proposed version generates waypoints to cover the narrow spaces while assuming appropriate morphology of the tiling robot in [17]. Several other researchers also worked on implementing the coverage path planning approaches in unmanned aerial vehicles, which can be used in various domains, such as agriculture, rescue operations, and surveillance [23,24,25].

An essential aspect of CACPP approaches is to breakdown the given area, which is defined as, a map. We can uncover the best paths if we can interpret the map in the best way. Numerous classical methods help in representing the environments. The basic strategy is to decompose the map into smaller regions known as sub-regions or cells. The traditional method is to divide the given area with simple shapes like triangles, trapezoids. The map can also be decomposed using the morse function [26], 3D data [27], landmarks [28], and graphs [29]. Researchers adopted different strategies for the map decomposition, as mentioned above. Another interesting approach is to implement the contact sensors for robots with sensor systems on-board. The current paper uses the grid-based decomposition as presented by the authors in [30,31]. Numerous algorithms are available that can enable us to divide an environment using different strategies like spanning trees [32], energy-aware algorithms [33,34], and neural networks [35], among others. The grid-based map decomposition algorithm is light-weight in terms of computational complexity for generating a coverage path.

The generic process of the CACPP for tiling robot consists of two separate processes as follows: Firstly, a tiling set is generated based on the robot morphology. This tiling set contains all the shapes that the robot can assume through reconfiguration. Coming to the hTrihex robot, this tiling set is generated based on the hexagonal polyomino tiling theory, and authors in the paper [16] presented several theorems with their proofs to tile a region with a hexagonal grid. Once the tilesets are generated, the robot, hTrihex, can move to different grid locations while reconfiguring the corresponding shape to assume the tile shape. In this way, the tiling theory algorithm can ensure that every nook-and-corner is covered by the robot. The scope of improvement here is that the traditional tiling theory algorithm generates tilesets that are not optimal. That means the robot has to assume shapes without any intelligence. Specifically, there can be instances where the robot can clean a particular area by reconfiguring once or not reconfiguring at all.

On the other hand, the tiling theory might suggest the robot to perform multiple redundant reconfigurations at every point. This type of implementation takes a toll on the robot’s energy consumption. Coming to the second process, the generated tilesets have to be connected with a proper trajectory. This can be achieved through conventional path planning algorithms like zigzag, spiral, and greedy search. Another side of the coin is that their performance is directly dependent on the environment’s shape and the obstacles involved.

As the kinematic performance of the tiling robot is also influenced by its surroundings, it is important to understand the uncertainties that might arise from the environment and or within the robot itself. The uncertainties from the environment can come in different forms such as the type of the flooring, elevation, ruggedness among others. The main important thing to focus on is the structural reliability of the platform itself. There can be uncertainties that might arise within the robot due to several reasons like wear and tear of the mechanisms, slippage of motor wheels, manufacturing tolerances among others. The error gain can be huge and it will impact the performance of the entire system. Reliability analysis of the robot is one of the important aspects to understand the error distribution from which we can perform fault analysis. Identifying the root cause and correcting it can increase the system reliability which in this case the accuracy of the robot to reach a given location without errors. Several promising methods [36,37,38] were suggested to estimate the reliability of a system with moving joints. The authors in the paper [36] proposed an accurate method of computing the system reliability of robotic manipulators. They utilized the principle of maximum entropy to present their innovative method that can evaluate the distribution of maximum positional error. The authors calculated a trajectory based on the approximate kinematics and the difference between the simulated trajectory and that required by the design and this is termed as the positional error. This parameter is computed over a sample data and the results give an error distribution through which the system reliability can be estimated. The authors in the paper [37] proposed to develop a rotational sparse grid (R-SPGR) method for statistical moment evaluation and structural reliability analysis that has enhanced accuracy and efficiency. Through this method they are collecting information about the probability distribution of the system response upon which the probability density function and failure probability of the system response can be estimated through saddlepoint approximation technique. They validated the system by taking several samples and also a practical example. The authors in the paper [38] proposed an improved moment-based method integrated with Lie-group theory, series expansion simulation, sparse grid Gauss-Hermite integration technique and chi-square approximation to estimate the time-dependent positional reliability of a system. This reliability is nothing but the probability of the positional error falling within a safe region over a certain time interval. This is an interesting method as this can estimate the reliability of the robotic system over a certain time interval with accurate results. In the current paper, for the ease of implementation, the uncertainties from the environment or within the robot are not considered.

To find the optimal trajectory for generated hexagon based tileset, instead of bluntly generating a path using the methods mentioned above, this problem can be assumed as a traveling salesman problem (TSP) that can generate a cost-efficient path. Nevertheless, it can be understood from the claims of the authors in the paper [39], the main problem with the TSP is the availability of an immense number of possible trajectory options, thus making it an NP-hard problem. This method is not feasible in the current case as the robot has to perform path planning and execute control decisions in real-time which is computationally expensive. Another strategy is to utilize evolutionary algorithms like the genetic algorithm (GA) or ant colony optimization (ACO) algorithm to solve this TSP within an acceptable time duration. On the other hand, the mentioned solution is highly dependent on the extraction of landmark features and cannot be scaled to any kind of environment. Optimizing this method also takes numerous iterations, which increments the computation cost, and the results can be sub-optimal if the optimization gets stuck at a local minimum.

To solve the issues posed by tiling based complete area coverage methods mentioned above, a robust strategy for the modified hTrihex robot is described in this paper. Literature shows a possible implementation of reinforcement learning to generate paths for robots in complex environments. Authors in the paper [40] implemented a reinforcement learning-based approach on the robot titled hTetro, that can shapeshift into seven shapes, in a square grid that can help the robot plan paths and change shapes simultaneously based on the environment layouts. The current paper explores the similar theoretical approach on modified hTrihex that moves in a hexagonal grid. Some other researchers used RRT based planners to find optimum positions. Authors in [41] proposed a new approach called deep reinforced learning tree (DRLT) based on the Deep Reinforcement Learning to estimate the deployment locations of mobile robotic sensor nodes, forming a wireless sensor network, for spatiotemporal monitoring. The idea is to find the locations where they can receive maximum information from the sensors. The parameterized sampling locations in the given space are modelled according to their spatiotemporal correlations which is subjected to constraints like field estimation error and information gain. The authors used a model-based approach with which the information gain can be calculated over an infinite horizon. Upon learning the effectiveness of the sampling locations, the DRLT advises the robotic sensors to avoid unnecessary sampling locations in the future iterations. Authors in [42] proposed a novel idea to plan paths in unexplored layouts. They performed a fusion of high-level global visiting order inference and low-level inter-regional planning. They implemented a deep reinforcement learning model to learn the common knowledge in office floor plans, and successfully generalized the model to new cases via defining a MDP over the heuristic graphs. They also used the next best value (NBV) algorithm as a waypoint selection method that can help in explorations. Authors in [43] propose a reinforcement learning-based model for path planning in a simple two-wheeled robot. They attempted to model the path planning problem as a state–action problem and derive the functions that can provide each state’s value. The authors claim that this method provides a more straightforward solution for robots with non-holonomic drive constraints. The paper [44] proposes an alternative for the existing best first search (BFS) and rapidly-exploring random trees algorithms, as they generate paths that are sharp and are not suitable for smooth navigation. Thus, the authors utilized the reinforcement learning-based Q-learning approach to automatically remove the paths that collide with the obstacles and thus produce smoother paths for seamless navigation. Authors in [45] proposed the application of double Q-network (DDQN) reinforcement learning to explore the path in an unknown environment dynamically. The authors claim that the Q-learning enhances the agent’s ability to plan the path in a dynamic environment with obstacle avoidance as the agent was trained in different environments. The paper [46] describes the application of reinforcement learning in space explorations. The authors trained the reinforcement learning on a quadruped ant-like robot with randomized reward function parameters. The agent was trained on the randomly generated waypoints, and these waypoints were then passed into the neural network for the corresponding action. The authors claim that this type of implementation would help the robots explore in unknown spaces.

The main contributions for the current paper are summarized as follows:

- *An RL Approach for CACPP:* Unlike the previous conventional tiling theory methods, a reinforcement learning-based deep learning model was developed to execute complete area coverage path planning by the modified hTrihex robot.
- *Reinforcement learning technique to enable the self-reconfigurable robot (modified hTrihex) to change shape and plan path simultaneously:* The model’s objective is to determine a path with less cost weight and a minimum number of morphology changes compared with the previously mentioned tiling theory approaches.
- *Simulation Studies:* Simulation studies were conducted to evaluate the performance of the proposed CACPP approach from different perspectives compared to conventional methods. The experimental results for different environments demonstrate the efficiency and scalability of the proposed approach.

The current paper is structured as follows: Section 2 illustrates the modified hTrihex platform, and the cost functions considered during the implementation. The cost function is directly dependent on the energy consumption and the robot action duration. The main reason for optimizing energy consumption is to enable the robot to work for a more extended period of time. Section 3 deals with the methodology and discusses the state environment representation, the network architecture, training algorithm, and the reward function utilized. Section 4 discusses the proposed algorithm’s performance against other approaches and its performance in various environments. Section 5 discusses the results and Section 6 provides the conclusion and future works for the current paper.

## 2. Modified hTrihex Reconfigurable Platform

**Figure 1:**
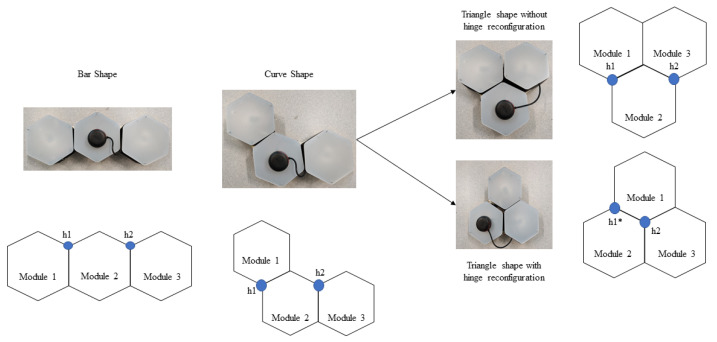
Modified hTrihex reconfigurable robot with three hexagon-based morphologies. (The h1* shows the new position of the hinge after hinge reconfiguration.)

**Figure 2:**
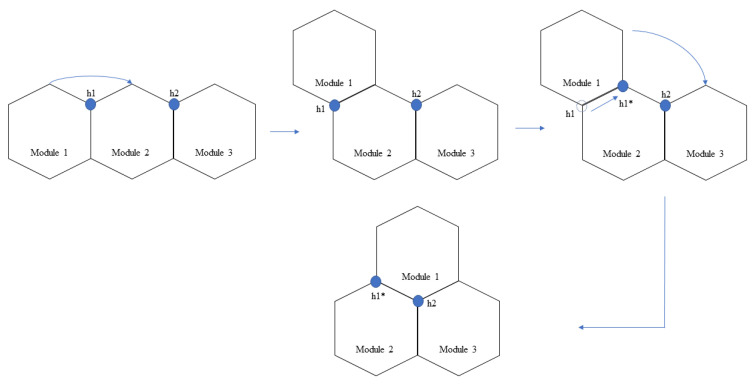
Modified hTrihex with a reconfigurable hinge joint. (The h1* shows the new position of the hinge after hinge reconfiguration.)

The modified hTrihex platform comprises three blocks named 1, 2, and 3, as shown in Figure 1. It is a bio-inspired design based on the honeycomb structure. It was considered that the modified hTrihex platform has two hinges on block 2, and the blocks 1 and 3 can rotate within an angular range of 0–2π/3 radians each to assume three different shapes namely, Bar, Curve and Triangle as illustrated in Figure 1. The current paper also assumes a design consideration of reconfigurable hinges. That means the hinges can move along one of the given edges of the hexagon block. There are two hinges, namely ‘h1’ and ‘h2’. In the current attempt, we consider that only hinge ‘h1’ has the ability to slide over one of the edges of block 2. The hinge reconfiguration and the shape that is assumed by the robot after the hinge reconfiguration are illustrated in Figure 2. The three configurations can help the robot to cover a given area. If we observe, with the hinge ‘h1’ reconfiguration, the block 1 can rotate a total of 4π/3 radians from Bar shape position to assume a triangle shape.

The modified hTrihex robot can perform three generic locomotion movements: Translation, Rotation, and Transformation. The kinematic modeling of the hTrihex robot is discussed in [47] which is similar to current modified hTrihex robot.

**Figure 3:**
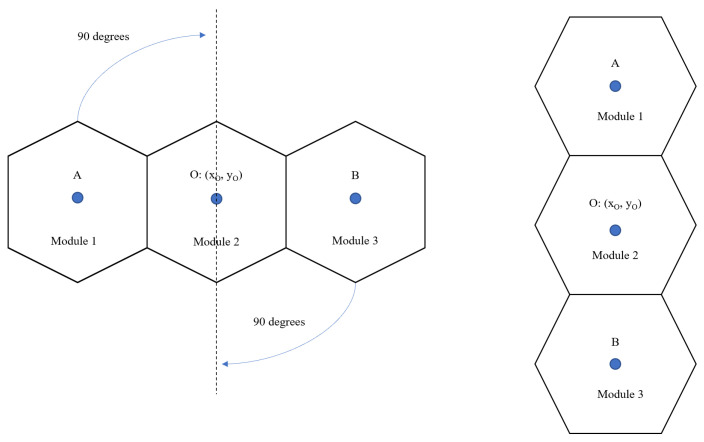
Illustration of a hTrihex orientation change.

In this paper’s context, we understand that every action the robot performs will have corresponding energy consumption. In terms of generating the path, the action space must be discretized, and it cannot be a combination of multiple motions. The cost function is modelled based on the total distance traversed by each block to perform a specific action and the weight of the corresponding block. This is considered as cost-weight. For cost-weight calculations, the robot’s total weight is considered to be 1.2 kg [16]. Here, the weight of each module is approximated to be 0.4 kg and is expressed by $m_{i}$, where *m* denotes mass and *i* denotes the index of the block. For instance, the cost-weight for translating from one point to another point in a given area is determined based on the Euclidean distance between the first point and the second point, which are source point ($x_{i}$,$y_{i}$) and destination point ($x_{j}$,$y_{j}$), multiplied by the weight of each block. Under the case of rotation and transformation, module 2 is considered to be fixed. Let us consider a situation where the robot has to make a rotation of π/2 radians. As per the previous assumption, let us fix the position of module 2. Then the cost of this motion attempt is given by Equation (2). The cost of the translation is given by Equation (1). This motion attempt as an example is illustrated in Figure 3. The cost-weight for the other movements are also computed in a similar way.

(1) $$C_{translation}=\sum_{i=A,B,C}m_{i}\times \sqrt{{\left(x_{i}-x_{j}\right)}^{2}+{\left(y_{i}-y_{j}\right)}^{2}}$$

(2) $$C_{rotation}=\pi /2\times \left(\sum_{i=A,B}m_{i}\times \sqrt{{\left(x_{i}-x_{o}\right)}^{2}+{\left(y_{i}-y_{o}\right)}^{2}}\right)$$

(3) $$C_{total}\left(x\right)=\left\{\begin{array}{cc} C_{translation},x\in \left(up,down,left,right\right)\; & \\ C_{rotation},x\in \left(clockwise,anti-clockwise\right)\; & \\ C_{transformation},x\in \left(Bar,Curve,Triangle\right) \end{array}\right.$$

**Figure 4:**
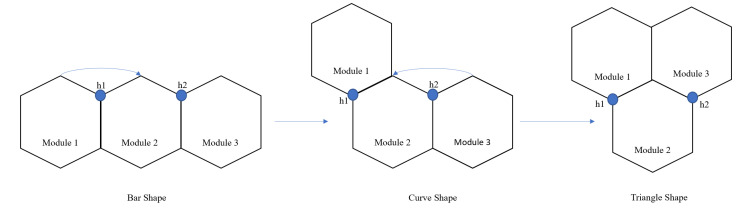
Illustration of hTrihex transformation changes.

The total cost-weight of every motion endeavour is given by the sum of translation, rotation, and transformation costs. The transformation cost-weights are determined based on the current shape and the target shape. As mentioned earlier, the current robot can assume three morphologies (bar, curve, and triangle). For a clear understanding, the transformation costs, without weight, to change from one shape to another shape is given in Table 1. To attain a specific shape transformation, the blocks undergo rotations concerning the hinges. The transformations are illustrated in Figure 4. Under the hinge reconfiguration assumption, the block 1 still need to move 2π/3 radians for two times to assume a triangle shape. This cost is equivalent to two blocks moving 2π/3 radians each to assume a triangle shape. For the ease of simulation, we neglected the cost of the hinge reconfiguration.

If the length of the side of a hexagon is L, then the diagonal length passing through that vertex will be 2L. Let us consider the instance where the robot has to transform its shape from Bar to Triangle. Module 1 and Module 3 must rotate an angle of 2π/3 radians each. The transformation cost for one module to rotate an angle of 2π/3 radians is 4πL/3. Therefore, the total cost for the transformation attempt from Bar to Triangle is 8πL/3. The technical implementation is usually that the real-world environments are perceived by the LiDAR sensor and converted into a discrete hexagonal grid of a fixed size. To ease the implementation, each grid block’s size is considered the same size as one module of the hTrihex.

## 3. Proposed Methodology

In the current paper, the path planning attempt for the modified hTrihex robot is modelled as a Markov decision process (MDP). As per the MDP, we have state information that addresses the robot’s shape, environment, and cleaning progress. Under the set of actions, we have four movements (up, down, left, and right), two rotations (clockwise, anti-clockwise), and three transformations (bar, triangle, and curve), as mentioned in the previous section. As per the MDP, the reward function based on the robot kinematic design has to be determined, which is discussed in the latter part of this section. We aim to determine the probability function that associates the current state (s) of the robot with an action (a) that results in a state (s’). Due to the various possible shape combinations and actions for different workspaces, it is not logical to calculate this function through the brute-force method. One possible method is to estimate this function through a deep learning architecture. The current paper utilizes a convolutional neural network-long short term memory (CNN-LSTM) model to perform the estimation.

**Figure 5:**
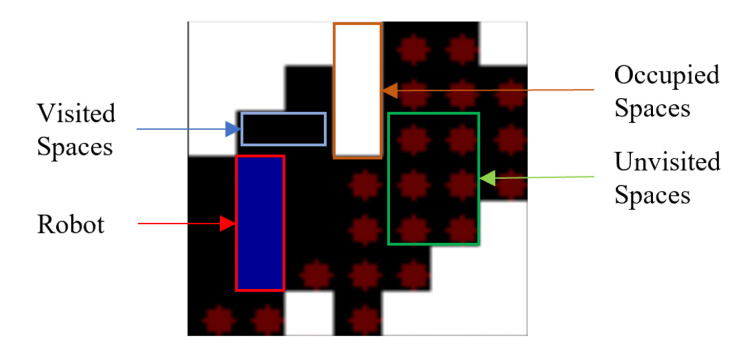
State representation.

The RL model’s training process involves the conversion of the state maps into images of size 70 × 70. These images contain specific feature information pertaining to the environment. Cells with white colour are considered as obstacles. Every empty cell is regarded as of two types: cleaned or yet to be cleaned. The cells that are not visited by the robot contain red diamonds, whereas the visited cells are empty, illustrating the fact that the robot had cleaned that particular cell. The robot is represented in blue colour occupying three cells in any instance. Using this type of discrete environment representation reduces the model’s computational complexity to perceive and understand the current state. A sample state environment space is illustrated as in Figure 5.

### 3.1. Decision Network

**Figure 6:**
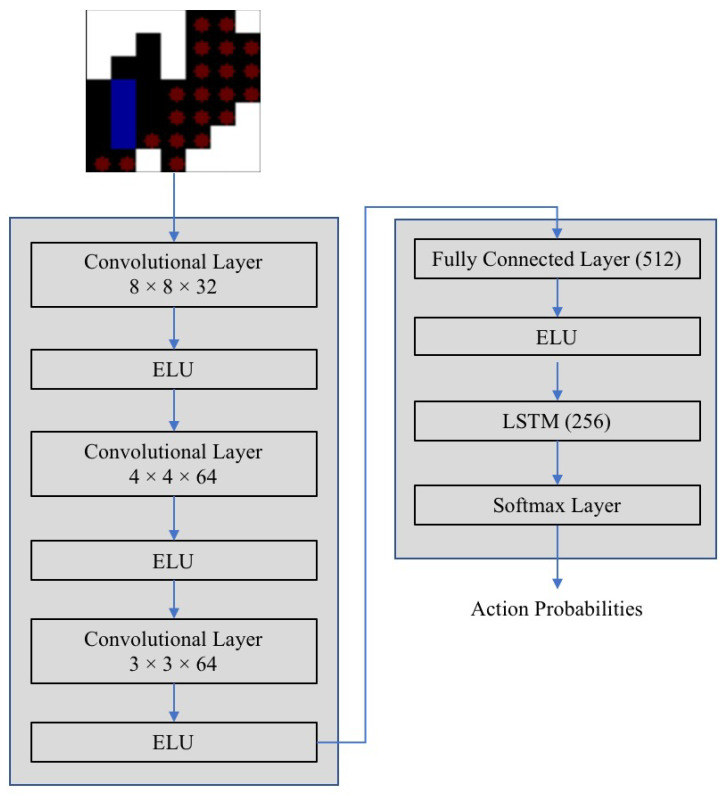
Decision network.

As mentioned in the earlier section, the execution of an action at any state is determined by the probability function which is decided by the CNN LSTM network. Figure 6. illustrates network architecture.

The architecture comprises of three convolutional layers of different kernel sizes to extract features from the state environment. To introduce non-linearity in the output, the exponential linear unit (ELU) activation function is considered in every layer. The function takes in the input *x* and outputs the same input *x* if *x* > 0, and if *x* < 0, then the input is fed through the expression $\alpha (e^{x}-1)$, where α is an ELU hyperparameter that is a scalar and controls the value to which an ELU saturates for negative inputs. The value of α is taken as 1. The exponential linear unit (ELU) activation function is given by Equation (4).

(4) $$g\left(x\right)=\left\{\begin{array}{ccc} & \alpha (e^{x}-1) & for\;x\leq 0\\ \; & x & for\;x>0 \end{array}\right.$$

The extracted features are then fed into the fully connected layer, which also has exponential linear unit (ELU) as the activation function, followed by an LSTM with softmax activation. The softmax function is given by Equation (5). The outputs of the softmax function are always bounded between the range [0,1] and the resultant probabilities add up to be 1, thus, forming a probability distribution. Here, *n* is the number of actions in action space and *x* is the value of the input from the previous layer.

(5) $$s\left(x_{i}\right)=\frac{e^{x_{i}}}{\sum_{j=1}^{n}e^{x_{j}}}$$

The LSTM network enables the model to learn and take necessary actions for covering a particular area with a specific shape and refrain the robot from visiting the already cleaned points.

### 3.2. Training the Decision Network

As mentioned in the previous section, the reinforcement learning (RL) method is used to train the network for decisions. There are numerous reinforcement learning algorithms as per the literature review [43], but only a few help train the agent in our case scenario. The current paper is composed of state and action pairs that result in a wide range of combinations. Hence, model-based techniques are not suitable for our case. This led to selecting the model-free methods. There are many model-free techniques, such as, consider Q learning. It is not suitable here because of the possibility of a large number of actions at any given state space. In this scenario, the goal should be to predict an action that results in the best rewards at any given state. This leads to another issue since the current scenario is a coverage problem; just selecting an action based on the immediate reward is not logical. This results in a situation where the action avoidance with not so large reward is given higher priority. This, in turn, results in not executing a complete area coverage, which is the ultimate goal of this paper. In this regard, we consider a policy gradient technique that looks into the corresponding future rewards as well. Such a technique results in improved area coverage efficiency.

The policy gradient models are brought up in as actor-critic model. It is a standard RL technique that relies upon the policy gradient approach. In short, it usually contains two parts: Actor and Critic. The actor’s function is to estimate a policy while the critic evaluates the policy’s decisions. It is analogous to a feedback system, where the actor’s training is based on the critic’s evaluation results. Even within the actor-critic model, there are two more policy variants: On-policy and Off-policy. One classic example of an on-policy method is A3C [48]. It is a base algorithm that executes a complete run before moving to the evaluation phase. Coming to the off-policy approach, it is advantageous over the on-policy method because of its sample efficiency and impressive exploration. In the current paper, with the complex state and action pairs’ existence, it is suitable to utilize an off-policy method for the above-mentioned actor-critic model. This type of actor-critic model implementation is known as the actor-critic with experience replay (ACER).

### 3.3. ACER Reinforcement Learning Algorithm

As mentioned in the previous section, ACER is an off-policy implementation of A3C. During the model’s training, the rewards, states, and actions are stored in a structure known as experience replay buffer. Specifically chosen experiences are selected from this stored buffer and are utilized in training the model’s policy. The utilization of marginal value functions helps in estimating the change in policy at each step. The marginal value policy gradient is given by Equation (6).

(6) $$g_{mar}=E_{S_{t},a_{t}}\left[\rho_{t}\times \nabla_{\Theta }\times \log\left(\pi_{\Theta }\left(a_{t}/S_{t}\right)\right)\times Q^{\pi }\left(S_{t},a_{t}\right)\right]$$

In the Equation (6), E is expectation, $\pi_{\theta }$ is a policy parameterized by Θ, $\rho_{t}$ is the importance weight, $s_{t}$ and $a_{t}$ are the state and action at a given time *t*. Optimization functions are necessary for any model. In this case, the ACER utilizes three optimizations for this function. Initially, the model uses the retrace q-value estimation technique, as given by Equation (7), to train the critic instead of standard q-value because of its impressive performance in reducing the bias while during the off-policy updates.

(7) $$\begin{array}{c} Q_{ret}\left(S_{t},a_{t}\right)=r_{t}+\left(\gamma \times \rho_{t+1}\times \left[Q_{ret}\left(S_{t+1},a_{t+1}\right)-Q\left(S_{t+1},a_{t+1}\right)\right]\right)+\left(\gamma \times E\left(S_{t+1}\right)\right) \end{array}$$

In the Equation (7), $s_{t}$ and $a_{t}$ are state and the action pair for which the reward $r_{t}$ is generated. *Q* is the standard q-value estimate, γ is the discount factor and E is the expected Q value. Coming to the other two optimizations, both of them are focused on the reducing the variance while training. The first one is responsible for reducing the impact of large ρ values on the final marginal value function. This is achieved through introducing a correction term and truncating the importance weight ρ. This correction term is added to Equation (6). The next optimization depends on the concept of trust region policy optimization during training. The trust region policy optimization (TRPO) basically employs the Kullback–Leibler (KL) divergence to estimate the loss that has to be optimized by a specific optimizer. ACER model modified the KL divergence implementation to improve its efficiency. Instead of measuring the KL divergence between policies before and after one update, ACER algorithm maintains the running average of the past policies and keeps the updated policy to not get deviated from this average.

### 3.4. Loss Optimization

Loss generated while training must be optimized. In the current implementation, the root mean squared (RMS) optimizer is utilized. It usually estimates the nodes’ weight at any time based on the gradient and exponential average while considering the current weights. The given Equations (8)–(10) are employed to update the weights in the network architecture.

(8) $$v_{t}=\beta v_{t-1}+\left(1-\beta \right)g_{t}^{2}$$

(9) $$▵w^{rms}=\frac{-\eta }{\sqrt{v_{t}+\epsilon }}\times g_{t}$$

(10) $$w_{t+1}^{rms}=w_{t}^{rms}+▵w^{rms}$$

Here β and ϵ are the hyperparameters while the η is the learning rate.

### 3.5. Reward Function

The agent learns efficiently with a proper reward function. This makes reward functions crucial in any RL training implementation. In the current paper, regarding CACPP, the maximum reward is awarded to the robot when it covers (cleans) an entire area. The convergence of the model is a higher priority objective. With such a vague reward function, the convergence can be difficult to achieve. Thus, with that consideration, the robot is rewarded whenever it covers a new tile. As mentioned in the previous sections, there is a specific cost for transformations, translations, and rotations. Each action’s corresponding cost is eliminated from the given reward as the robot performs such actions to cover a new tile. In that case, if the robot covers only the tiles which are already visited, then the reward will be negative. The idea of negative reward prevents the robot from re-visiting the same tiles, again and again, thus avoiding the robot from getting stuck in a loop. The reward assigned for visiting a new tile is more than the cost of the action. This enables the robot to perform multiple actions to cover a new tile. Regarding the negative rewards, a large negative reward will be assigned if the network suggests an illegal move. In this case, the movement will not be executed. For instance, suggesting an action that might end the robot in collision with any obstacle will attract a massive negative reward. Like, in the situation where the rooms are bounded with the walls, then any action that puts the robot in collision with any kind of obstacle will attract penalty. This also includes misjudging a transformation in any inadequate space. The effective reward *R* is given by Equation (11).

(11) $$R\left(a_{t}\right)=\left(n_{new}\times r_{new}\right)+\left(status\times r_{final}\right)-C\left(a_{t}\right)-\left(illegal\times C_{illegal}\right)$$

Here, $n_{new}$ is the number of new tiles visited, $r_{new}$ is the reward assigned for visiting a new tile, status is a Boolean flag which expresses whether the entire coverage task is completed or not, $r_{final}$ is the reward awarded for completing the coverage task. C($a_{t}$) is the cost incurred for a corresponding action at a time *t*. $C_{illegal}$ is the negative reward assigned to the robot if it takes an illegal action at a certain time step. All these reward parameters are assigned with values as per the experimentation.

### 3.6. Hexagonal Coordinate System

Square grids are the most common choice for implementing path planning and area coverage algorithms. In this paper, for the hexagonal morphological robot, a hexagonal grid is needed. Concerning the implementation of RL frameworks, the coordinate system of the hexagonal system is not direct and common as compared to a square grid. As mentioned in [49], a hexagon is a 6-sided polygon with six corners and sides. The corners are shared with three other hexagons, while the edges are shared with one adjacent hexagon. To ease the RL implementation during training and validation, in the current paper, the axial coordinate system was chosen.

The axial coordinate system is basically a skewed coordinate system. The axial coordinate system is based on the cube coordinate system. The Cube coordinate system comprises three primary axes. The hexagons are formed by partitioning the cube with a diagonal plane given by equation *x* + *y* + *z* = 0. Each cube is represented by three coordinates and traversal in a particular axis is made possible by keeping a constant value in one axis. In this regard, we can remove the redundancy of using all the three coordinate values to traverse in a particular direction. This results in the axial coordinate system where the *y*-axis was made constant. The reason to choose the axial coordinate system is its ease of implementing the vector operations on the coordinates.

The axial coordinate system offers a simple solution to help implement the hexagonal grid in a 2D array format. However, the only catch is that this leads to cell wastage, which is expected over all the other hexagonal coordinate systems. To represent a hexagonal grid in a 2D array, specific cells need to be declared with null values, which are typically not accessible. For the ease of simulation implementation, in the current paper, we adopted the square grid structure, as represented by the author in [49], for the robot to move, where the null places are considered to be obstacles, which the robot cannot access. In this way, we attempted to mimic the hexagonal grid in the axial coordinate system using the square grid. In short, for ease of simulating, the robot moves in the square grid that represents the hexagonal grid.

## 4. Experimental Results

The current experiment aims to compare the efficiency, time (to generate the path), and the cost-weight of the path generated with different methods as mentioned in paper [16] and the current technique. The area coverage is not considered as an attribute of interest because of the underlying assumption that the reconfigurability of the robot enables it to cover the whole area. Thus, this brings us to comparing previously mentioned metrics. The cost-weight for each motion attempt is computed with the help of Equation (3). The experimentations from [16] are executed on a map of size 5 × 8. The cost of the entire endeavour and the time taken to execute the path is measured and compared. In the current paper, the comparison is made against the traditional titling based path planning strategies, as mentioned in [16]. In paper [16], the tilesets are obtained first, and then a path planning algorithm is employed to connect all the tilesets with an optimized path. The conventional genetic algorithm (GA) and ant-colony optimization (ACO) algorithms are commonly used to generate paths or trajectories.

In the current paper, coming to the hyperparameter tuning, they were tuned based on the experimental trials. After testing with 40 random environments mimicking different real-world space layouts, we arrived at the best hyperparameter values. The optimizer function’s learning rate was decided to be 0.0001 with a β of 0.99. In the ACER algorithm, the discount factor γ is kept at 0.9 with an experience reply buffer of size 20,000. Another critical aspect of making the model converge is to ensure that the coefficients in the reward function are adequately set. For cost computations, the size of the block and one grid cell are considered to be the same. The reward for covering the entire area is set as 20,000. The penalty for making a wrong move under any situation is set as 200. The reward for choosing the same shape as previous is kept as 200. All the obtained rewards are scaled-down by a factor of 400, and this combination of values proved to enable the model to converge for the given range of layouts.

### Experimental Results in the Simulation Environment

The current method covers the entire given area without leaving any grid cell uncleaned. We selected a few random environments from the set of total environments to validate the current model’s efficiency. The problem with the tiling based theories is that they have to adhere to certain theorems and axioms, as mentioned in [16] for tiling the given area. Thus, it cannot be expanded to every environment. Thus, the environment has to be specific. In the current approach, the robot trajectory and the transformations are decided simultaneously, thus allowing it to be a faster implementation.

**Figure 7:**
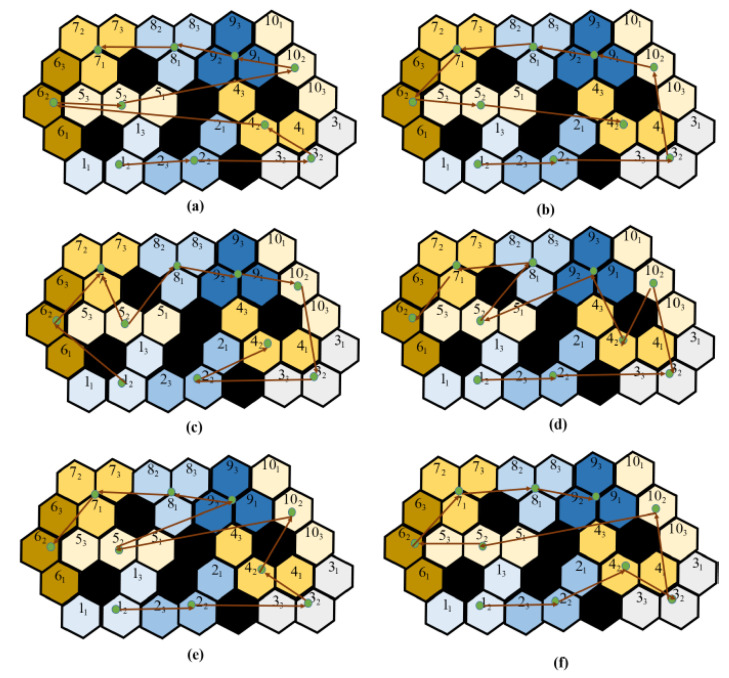
The trajectory comparisons of the complete area coverage path planning (CACPP) approaches as provided by [16]: (**a**) zigzag scanning (cost weight = 93.33); (**b**) spiral scanning (cost weight = 94.37); (**c**) random search (cost weight = 84.1); (**d**) greedy search (cost weight = 81.36); (**e**) GA (cost weight = 61.99); and (**f**) ACO (cost weight = 61.35).

The performance comparison is made against the theory-based tiling plans and the current model for the given layout space, as shown in Figure 7. The black grid cells at the centre of the region signify the obstacles. As shown in Figure, the zigzag model connects the tile pieces in a row/column fashion. The spiral model connects the tile pieces from the ultimate layer to the inner layers. As compared to these models, the random search and the greedy search model have little advantage in terms of generating a cost-effective path, but still, they fail when compared with the paths generated by genetic algorithms and the ACO algorithm. Though the zigzag model and the spiral model have lower computational complexity, the path’s efficiency is highly dependent on the type of the environment. The greedy algorithm has a high chance of getting stuck at the local optimum because it moves to the next step based on the given constraints. Approaching the current problem of connecting all the tilesets with an efficient path can be considered a traveling salesman problem (TSP). GA and ACO algorithms can be employed to find an efficient path. Both algorithms perform well, but their performance is hindered by the efficiency of the tiling theories. The tiling theory always tries to fill up the empty spaces with the most suitable transformation. If the trajectory generating algorithm does not consider the transformation and rotational costs, then the results would be sub-optimal. With the addition of obstacles, these costs incurred will be high as the robot sometimes has to assume multiple transformations or rotations to traverse around an obstacle. The Figure 7 shows the trajectories generated by various algorithms.

**Figure 8:**
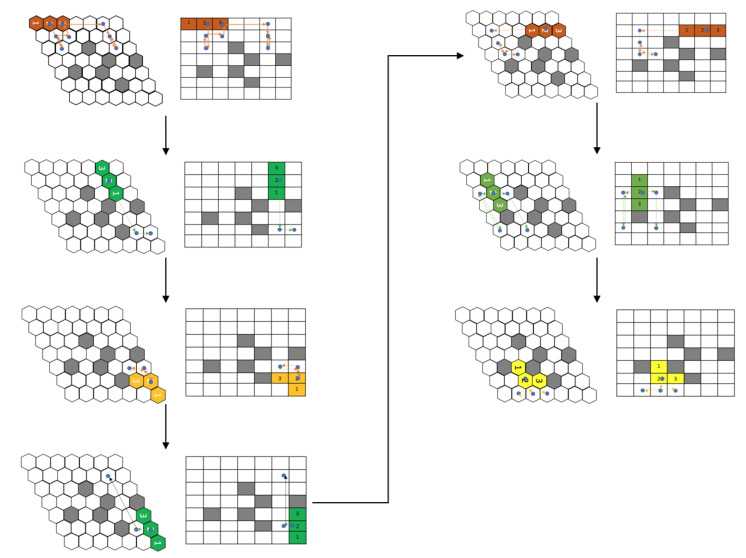
Simulation of a path as proposed by current approach for a similar obstacle environment as given in Figure 7.

The current model is trained against the same obstacle conditions, as presented in [16]. As the trained RL agent was deployed in a square grid, to enable free movement of the robot in the hexagonal grid, we consider the hexagonal grid with rhombus outline of grid size 7 × 7. Figure 8 shows the path and the shapes assumed by the modified hTrihex robot under a similar obstacle environment, as shown in the paper [16]. The blue dot signifies the second block and the lines represent the robot’s path under a specific shape transformation. To ensure a fair comparison against the algorithms mentioned in the [16], we used ν as a comparison parameter, which is the ratio of cost-weight and the number of empty cells in the given environment.

**Figure 9:**
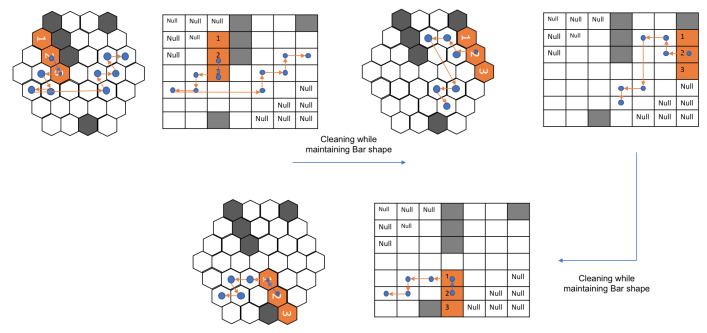
The first example of a path generated using the proposed approach.

Let us consider the map in Figure 9. In the first map, the model generated the path without any transformations required. The blue dots represent block 2 of the robot, and the orange lines show the path that the robot pursued based on the model output. In this first map, the robot was able to cover all the cells without any need for transforming into other shapes.

**Figure 10:**
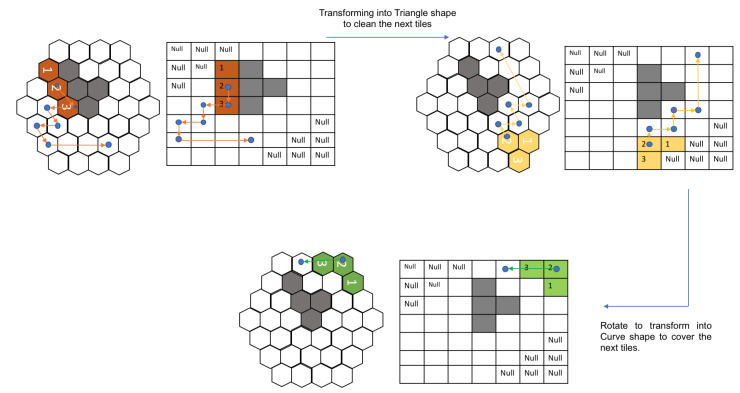
The second example of a path generated using the proposed approach.

In the next map, as shown in Figure 10, the robot first covers the given area with the Bar shape. The path taken while being in Bar shape is represented by the orange lines while the blue dot still signifies the block 2 of the robot. Once the robot reaches the point, as shown in Figure 10, second sub-figure, it assumes a Triangle shape to cover the next cells. This triangle shape is achieved with the help of reconfiguring hinge design consideration, as mentioned earlier. This new shape is represented in yellow, and the path that the robot adopted while being in the triangle shape is shown with yellow lines. As shown in Figure 10, third sub-figure, the robot realizes that it can access the remaining cells only in Curve shape. The robot rotates in the clockwise direction and this rotation in the square grid signifies the robot’s rotation and shape transformation into a Curve shape as per the hexagonal grid. With this shape, it covers the remaining cells, thus achieving 100% area coverage. As mentioned earlier, the traditional tiling theory algorithm requires a particular number of free spaces, as mentioned in paper [16] to generate the tilesets, and also the shape transformations are to be decided prior to the execution. However, in the current implementation, the trained model decides the shape required to transform based on the obstacle and environmental layout.

**Figure 11:**
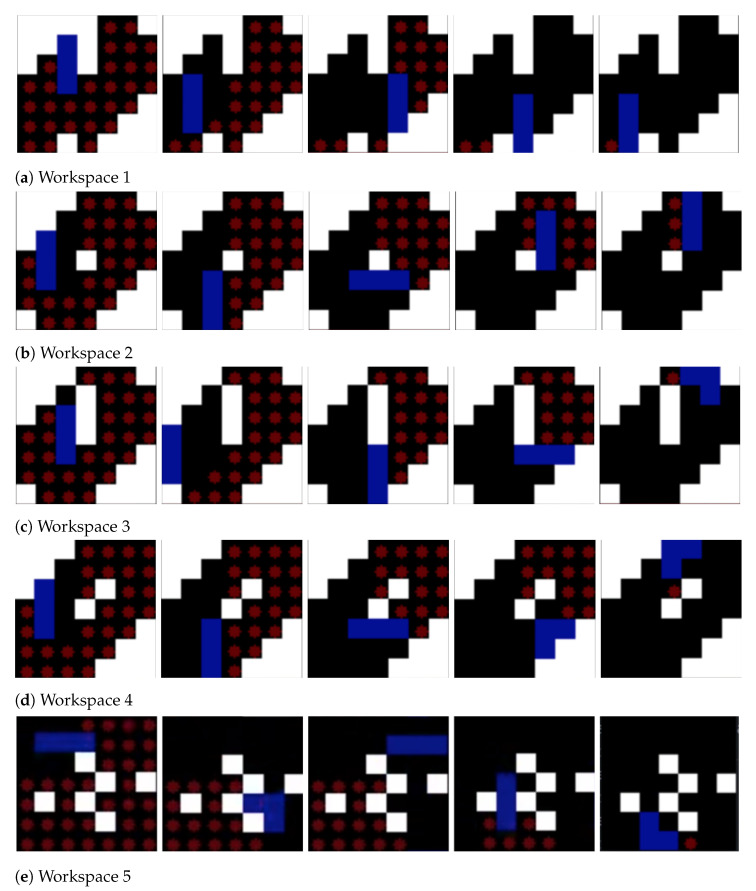
Paths adopted by the robot under different workspace arrangements in a simulation.

Figure 11 illustrates the sequence of actions that the robot assumed to clean the given spaces under different layout conditions. The model prefers to minimize the shape transformations as much as possible. For complex layouts, the model assumes shape transformations while monitoring the cost incurrence.

Table 2 shows that the current method resulted in a path with cost-weight of 59.52 Nm and with a path generation time of 0.314 s. The lowest ν value for the proposed method shows that the current method performed better as compared to the other traditional approaches. The proposed method generates a better cost-weight path (59.52 Nm as compared with ACO’s 61.35 Nm) and quicker (0.314 s compared to ACO’s 1.22 s). The running time signifies the time taken for the path to be generated by the algorithm. In our case, the time taken for the path generation is 0.314 s. However, as the current implementation is based on neural networks, there will be an overhead load time which can be taken care once every time the robot boots up. Thus, the RL based method saves time by simultaneously performing path planning and morphology changes based on the environment layout, unlike the traditional tiling theory-based path planning approaches.

## 5. Discussion

**Figure 12:**
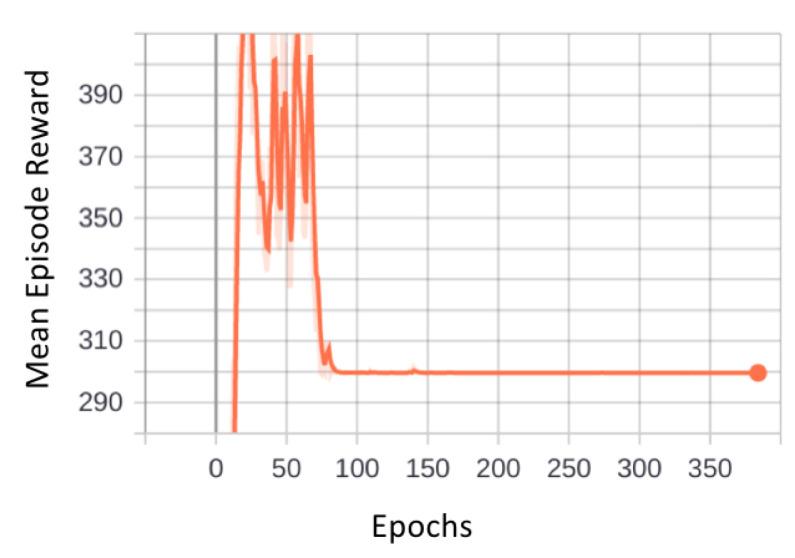
Illustration of mean episode reward vs. epochs.

The graph in Figure 12 illustrates the trend of mean episode rewards with reference to number of epochs. In each simulation, the robot must cover the entire area within a certain number of steps. We have used 10 parallel environments. This means that in 1 epoch there will be 10 episodes, and if we observe the graph, we can see that the convergence started from around epoch 85. That means it took the model 10 times 85 episodes, which is 850 episodes to get converged. After the end of every epoch, the policy is updated with new weights. Initially, the reward values were fluctuating, depicting that the robot was exploring the layout with different paths. Then, once the robot learned a suitable action at a particular state, the reward values started to arrive close to the true reward value, leading the model to convergence.

We have tested different hyperparameter combinations and found out that the combination that was mentioned previously in Section 4 resulted in convergence. Initially, we found that for some of the layouts the model got stuck in a local minimum. To avoid that, we had to go with a lower learning rate. This is an important aspect because if we keep a very low learning rate then the model might take a lot of time to find the global optimum. In our case, the learning rate 0.0001 gave the best results. Maybe with more complex and large layouts, the robot might take more time and computational memory requirements to sample more number of batches to better update the policies resulting in maximized rewards.

In the current paper, we have not considered unexplored layouts. Our model trains on the known layouts and then comes up with the suitable state–action pair that will maximize the reward values. As mentioned in the paper [42], we can use a systematic model to estimate the action in unexplored layouts as well, which will be considered as part of our future works. As mentioned in paper [41], using a model-based approach gives stable results but also results in increased computation complexity. In our case, we wanted to provide a simple method to cover the entire area as long as we are reducing the cost function and maximizing the rewards. Sufficient literature investigation has to be done to apply the state-of-the-art planning algorithms like rapidly exploring random trees (RRT) to tiling theory-based shape shifting robots. This can be taken up as a future work as well.

## 6. Conclusions

The complete area coverage path planning is a vital task that has to be catered by a cleaning robot. Efforts were driven in the current paper to propose a CACPP strategy based on a reinforcement learning framework. Workspaces with different layouts were generated, and the reinforcement learning agent, which was the modified hTrihex robot in our case, was trained over those layouts, thereby proving the robustness of the model. The simulated experimental results show that the robot could cover the whole layout with minimal rotational and transformational costs. Upon comparing the results with the conventional tiling strategies, we found that the proposed method offers the path with a better cost weight value. The proposed method is also capable of providing the best morphology based on the given layout. Unlike the conventional approach, where the layout is segmented into tilesets based on the tiling theory and then connects all the points through the optimization algorithms, the current approach simultaneously performs the shape changing and the path planning, thereby generating a less cost path in a quicker time period. The future work includes studying the robots’ decisions in partially unexplored environments and also exploring the multi-hinge reconfiguration. Another aspect of future work will be to convert the action space from discrete to continuous to command the motors in a continuous fashion, which will help in a real-time implementation.

## Figures and Tables

**Table 1:** Transformation cost ($C_{transformation}$) values. Column headings represent the current shapes. Row headings are the goal state.

	Bar	Triangle	Curve
Bar	-	8πL/3	4πL/3
Triangle	8πL/3	-	4πL/3
Curve	4πL/3	4πL/3	-

**Table 2:** Performance comparison of CACPP strategies in a simulated environment given in Figure 7.

Approach	Total Cost	Running	ν
	Weight (Nm)	Time (Second)	
Zigzag	93.33	0.01	3.11
Spiral	94.37	0.05	3.14
Random Search	84.1	31.34	2.80
Greedy Search	81.36	35.25	2.712
GA	61.99	1.25	2.066
ACO	61.35	1.22	2.045
Proposed RL Method	59.52	0.314	1.38

## Data Availability

Not applicable.
